# Two-Dimensional Co_2_S_2_ monolayer with robust ferromagnetism

**DOI:** 10.1038/s41598-017-16032-x

**Published:** 2017-11-22

**Authors:** Yun Zhang, Jingman Pang, Meiguang Zhang, Xiao Gu, Li Huang

**Affiliations:** 1 Department of Physics and Information Technology, Baoji University of Arts and Sciences, Baoji, 721016 China; 2 Department of Chemistry and Chemical Engineering, Baoji University of Arts and Science, Baoji, 721016 China; 3 Department of Applied Physics, Chongqing University, Chongqing, 400044 P.R. China; 4 Department of Physics, Southern University of Science and Technology, Shenzhen, Guangdong, 518055 China

## Abstract

Design and synthesis of two-dimensional (2D) materials with robust intrinsic ferromagnetism is highly desirable due to their potential applications in spintronics devices. In this work, we identify a new 2D cobalt sulfide (Co_2_S_2_) material by using first-principles calculations and particle swarm optimization (PSO) global structure search. We show that the 2D Co_2_S_2_ is most stable in the litharge type tetragonal structure with space group of P4/nmm. The elastic constants, phonon spectrum, and molecular dynamics simulation confirm its mechanical, dynamical and thermal stability, respectively. It is also found that Co_2_S_2_ monolayer is a ferromagnetic metal with a Curie temperature up to 404 K. In addition, we propose a feasible procedure to synthesize the Co_2_S_2_ monolayer by chemically exfoliating from bulk TlCo_2_S_2_ phase.

## Introduction

Over the past decade, atomically thin two-dimensional (2D) materials have been extensively studied because they have many unique and fascinating physical and chemical properties compared to their bulk counterparts. Many efforts have been devoted to searching new 2D materials since the first report of the successful fabrication of graphene by Geim and Novoselov in 2004^1^. Recent theoretical and experimental studies have shown that many more 2D materials can be synthesized, such as hexagonal boron nitride (h-BN), transition-metal dichalcogenides (TMDs), metal oxides, phospherene, silicene, germanene, and stanene, and all are under intensive investigation for their potential applications in nanoelectronic and optoelectronic devices^2–8^.

Among those novel 2D materials, TMDs, TMDs, a honeycomb structure with single or few atomic layers, have attracted a lot of interest owing to their diverse electronic properties with semiconducting, metallic, or superconducting states^9–12^. By using the micro-mechanical cleavage method, Novoselov *et al*.^13^ successfully synthesize the single layer MoS_2_. Following the Novoselov’s pioneering work, various TMDs, including WS_2_, TiS_2_, TiS_2_, TiSe_2_, MoSe_2_, TaSe_2_, NbSe_2_ and NiTe_2_, were gradually unraveled^14–24^. However, most of the pristine TMDs are intrinsically nonmagnetic, which hinders the utilization of these novel 2D nanosheets in the fields of spin-related nano-devices. Considerable efforts have been made to induce magnetism into these materials. For example, ferromagnetic characteristic has been detected in the MoS_2_ nanoribbons, which is attributed to the presence of zigzag edge^25,26^. Zhou *et al*. showed that the magnetism of MoS_2_ sheets can be tuned by embedding transition-metal atoms^27^. Chemical functionalization with hydrogen or fluorine is also an effective route to modify magnetic properties of TMDs^28–32^. Although these methods can induce magnetic behaviors in the 2D TMDs, it is difficult to control the edge structure and the doping/adsorption precisely in experiments. Therefore, developing a new type of TMDs materials with intrinsic magnetism in its pristine form is extremely urgent.

Recently, some ferromagnetic 2D monolayers containing 3d transition metal have been uncovered, such as FeC_2_, CrN, δ-FeOOH and MnS_2_^33–36^. Co is a typical 3d transition metal which has a Curie temperature Tc = 1405 K. Owing to the variable valence of Co, cobalt sulfides have diverse stoichiometric compositions such as CoS, CoS_2_, Co_9_S_8_, and Co_3_S_4_^37–45^. Their thermal stability and electronic conductivity are usually better than other metal sulfides and have been considered promising materials in many fields. Nevertheless, to our knowledge, there has been no report about 2D monolayer of cobalt sulfides. In this work, using particle-swarm optimization (PSO) techniques combined with first-principles calculations, we firstly predict a new 2D monolayer composed of cobalt and sulfur atoms, Co_2_S_2_. The calculated results reveal that the Co_2_S_2_ nanosheet is both dynamically and thermodynamically stable, and is a ferromagnetic metal with estimated Curie temperature up to 404 K. Finally, we propose a possible way to synthesize it by chemical exfoliation from its parent three-dimensional (3D) TlCo_2_S_2_ crystal. Our finding greatly enriches the 2D families of transition metal sulfides.

## Results

**Figure 1:**
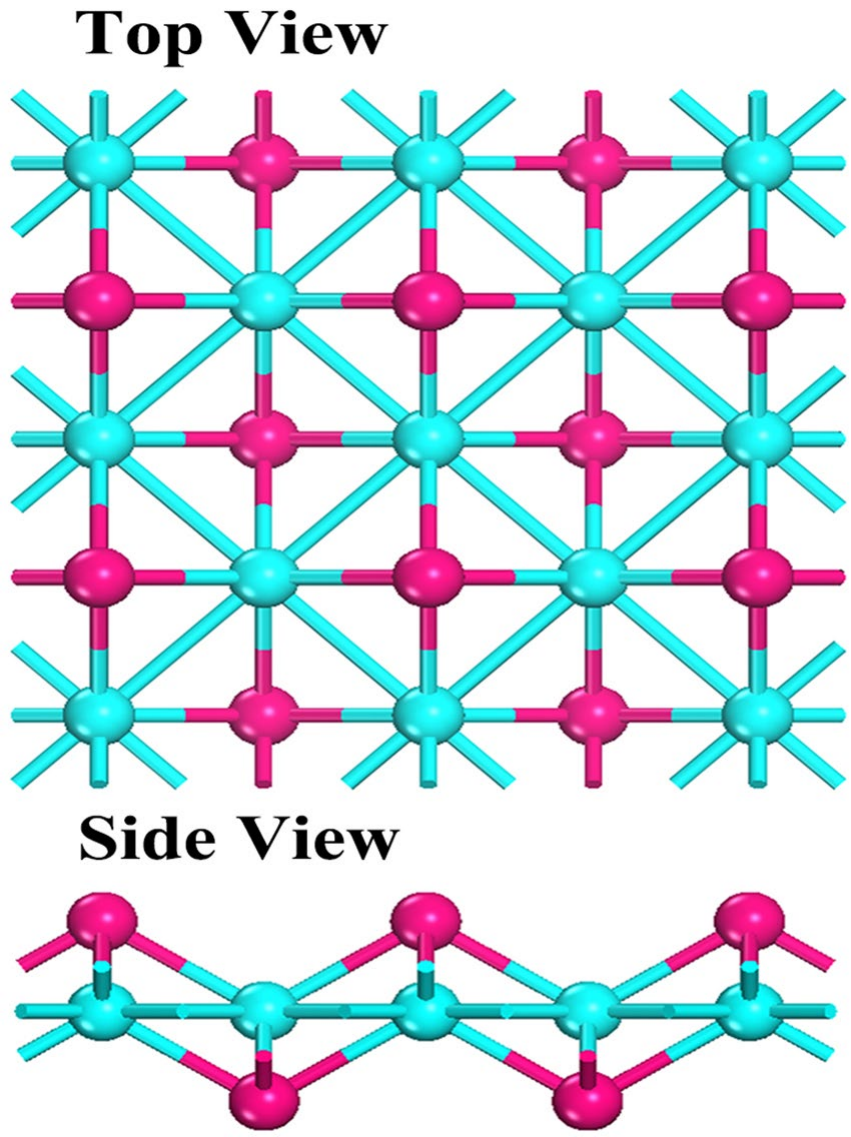
The top and side view of the 2D tetragonal structures of Co_2_S_2_. Purple and blue balls represent S and Co atoms, respectively.

The most stable structure of 2D cobalt sulfide is identified after 30 generations of searching. Figure 1 presents the top and side view of the optimized structure of Co_2_S_2_ monolayer, which crystallizes in the tetragonal space group, P4/nmm (No. 129), with each Co atom bonding with four S atoms and four Co atoms, and each S atom binding with four Co atoms. The unit cell of 2D Co_2_S_2_ monolayer contains two Co and two S atoms, and the optimized lattice parameters a = b = 3.638 Å. The structure of Co_2_S_2_ can be viewed as a single-layer PbO-type structure, in which cobalt atomic layer is sandwiched between the top and bottom sulfur atomic layers. The thickness of Co_2_S_2_ monolayer is 2.40 Å, and the bond lengths of Co-S and Co-Co are 2.182 Å and 2.572 Å, respectively.

To evaluate the stability of Co_2_S_2_ monolayer, we first calculate the average cohesive energy: $${E}_{coh}=(2{E}_{Co}$$ $$+2{E}_{S}-{E}_{C{o}_{2}{S}_{2}})/4$$, where $${E}_{Co}$$, $${E}_{S}$$, and $${E}_{C{o}_{2}{S}_{2}}$$ are the total energies of Co atom, S atom and one Co_2_S_2_ unit cell, respectively. The Co_2_S_2_ monolayer has a cohesive energy of 4.69 eV/atom, which is comparable to that of silicene (3.71 eV/atom), Cu_2_Si (3.46) and Be_2_C (4.86 eV/atom) at the same theoretical level^46,47^. The relatively large cohesive energy suggests that the Co_2_S_2_ monolayer is a strongly bonded network.

The key criteria for mechanical stability of a crystal are that the strain energy must be positive, which for a mechanically stable sheet would satisfy the following criteria^48,49^: C_11_ > 0, C_12_ > 0, C_44_ > 0, C_11_-C_12_ > 0. The 2D elastic constants are calculated to be: C_11_ = 106.2 N/m, C_12_ = 37.5 N/m, and C_44_ = 34.4 N/m, indicating that the Co_2_S_2_ monolayer has robust mechanical stability.

**Figure 2:**
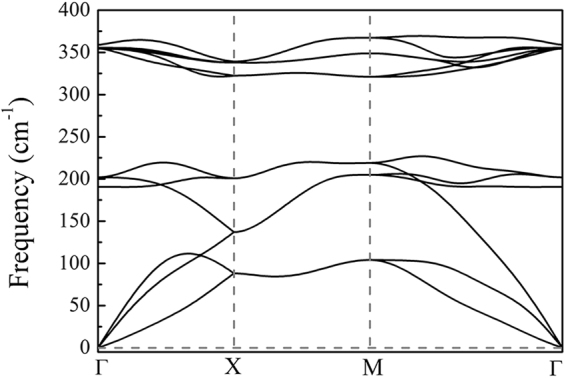
Calculated phonon dispersion along the high symmetry directions for the Co_2_S_2_ monolayer.

**Figure 3:**
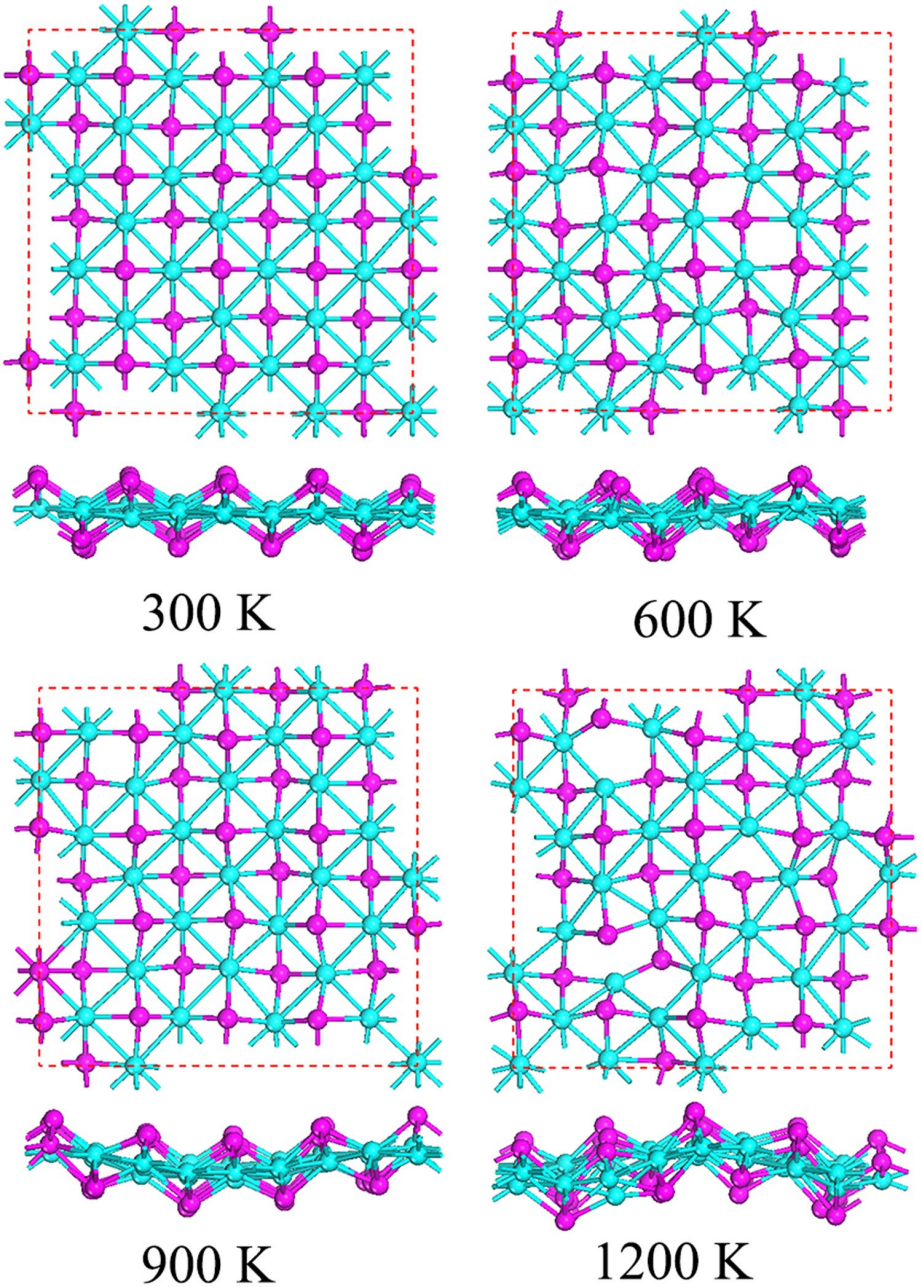
Structure snapshots of Co_2_S_2_ monolayer for AIMD simulation from 300 to 1200 K.

To further verify the structural stability of Co_2_S_2_ monolayer, we then calculate the phonon dispersion along the high-symmetry lines in the first Brillouin zone by using the Phonopy code. As shown in Fig. 2, the phonon spectrum shows no negative frequency in the whole Brillouin zone, which suggests Co_2_S_2_ monolayer is a stable phase without any dynamical instability. We also perform Ab initio molecular dynamics (AIMD) simulations to estimate the thermodynamical stability of 2D structure. Structure snapshots of Co_2_S_2_ monolayer taken at the end of each simulation are shown in Fig. 3. The results show that Co_2_S_2_ monolayer can maintain its structural integrity even up to 900 K. However, at the extremely high temperature of 1200 K, the planar structure is disrupted, indicating that 2D Co_2_S_2_ monolayer has good stability above the room temperature.

**Figure 4:**
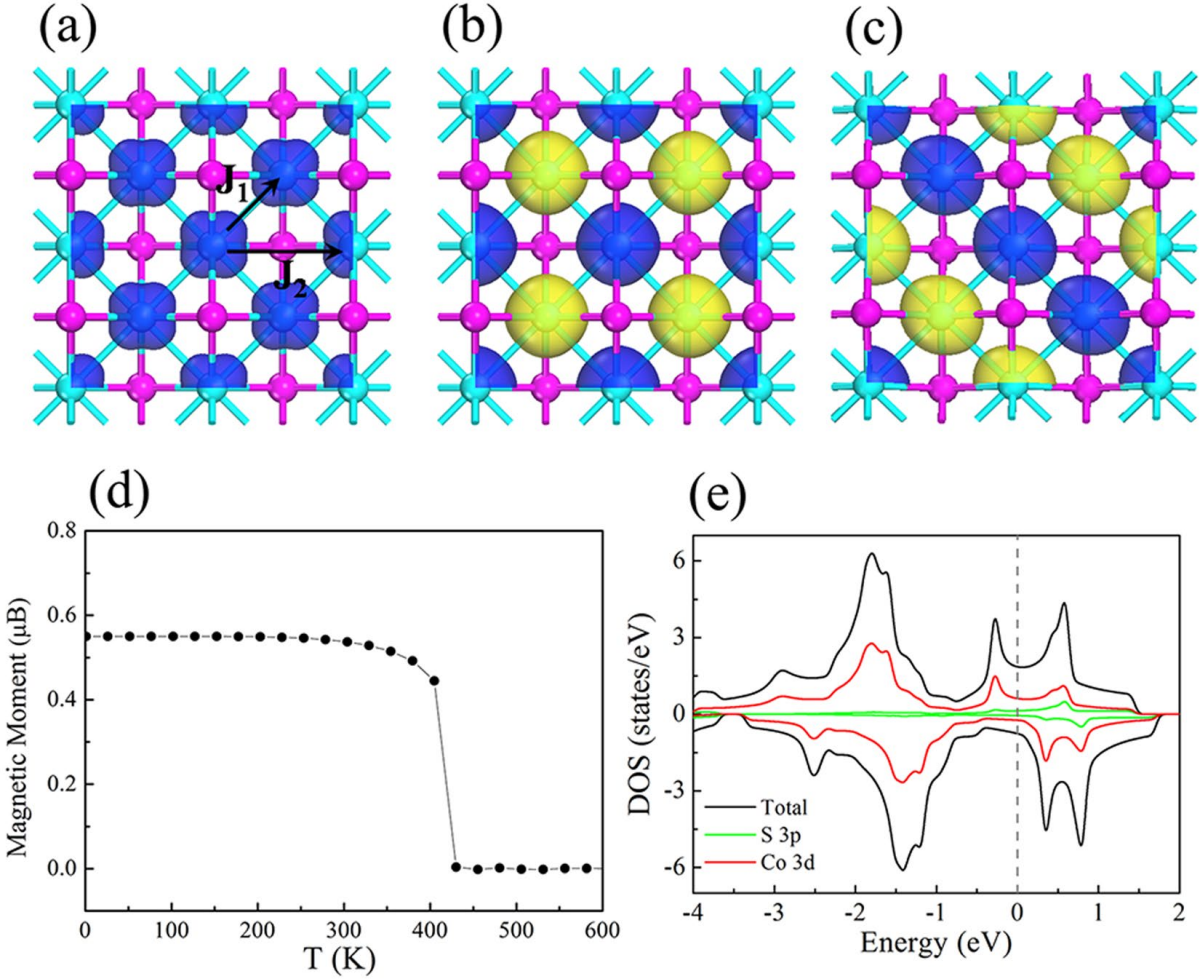
Spin density isosurface with a value of 0.01 e/Å3 for the FM (**a**), AFM1 (**b**) and AFM2 (**c**) coupling configurations of the Co_2_S_2_ monolayer. Blue and yellow indicate the positive (spin up) and negative (spin down) value, respectively. (**d**) Variation of the magnetic moment per Co atom with temperature. (**e**) The total DOS and PDOS for Co’s 3d and S’s 3p orbitals.

With the confirmed stability of the optimized monolayer Co_2_S_2_, we now turn to study the magnetic properties of Co_2_S_2_ monolayer. To explore the preferable magnetic ground state of Co_2_S_2_ monolayer, we construct three different initial magnetic configurations (i.e. FM, antiferromagnetic-1 (AFM1) and antiferromagnetic-2 (AFM2) states) in a 2 × 2 supercell, as shown in Fig. 4(a–c). The FM configuration is found to be 528 and 334 meV lower in energy than the AFM1 and AFM2 configurations per supercell, respectively, clearly indicating that FM state is the ground state of the Co_2_S_2_ monolayer, with a magnetic moment of 0.53 μB and 0.02 μB on each Co and S atom, respectively. We further calculate the magnetic anisotropy energy (MAE), and find that the easy axis is perpendicular to the c-axis with a MAE of 0.17 meV per Co atom.

To gain an insight into the magnetic properties of Co_2_S_2_ monolayer, we also calculated the total density of states (DOS) and the atomic site projected density of states (PDOS). As shown in Fig. 4(e), Co_2_S_2_ monolayer is a FM metal. We find that Co 3d orbitals have a significance contribution to the DOS around the Fermi level. There is noticeable hybridization between Co 3d states and S 3p states in both the spin up and down channels near the Fermi level, which demonstrates that the S 3p orbitals play a key role in the FM coupling of the Co_2_S_2_ monolayer.

Considering the practical application of 2D Co_2_S_2_ monolayer, it is quite interesting to know if its Curie temperature is comparable to or higher than room temperature. To this end, we use Monte Carlo simulation based on a simplified Ising model, $H=-\sum _{i,j}{J}_{i,j}{M}_{i}\cdot {M}_{j}$, where *J*_*i*,*j*_ is the nearest-neighbor exchange parameter and M is the local magnetic moment of Co atom. For simplicity, only two types of interactions in the Ising model are taken into account, i.e. the nearest-neighbor exchange parameter (*J*_1_) and the next-nearest-neighbor exchange parameter (*J*_2_), as shown in Fig. 4(a). Thus the expression for the exchange parameters for our system are ${J}_{1}=\frac{{E}_{AF{M}_{1}}-{E}_{FM}}{32{M}^{2}}$ and ${J}_{2}=\frac{2{E}_{AF{M}_{2}}-{E}_{FM}-{E}_{AF{M}_{1}}}{64{M}^{2}}$, where ${E}_{FM}$, ${E}_{AF{M}_{1}}$ and ${E}_{AF{M}_{2}}$ represent the total energy of FM, AFM_1_ and AFM_2_ states, respectively. The factor (1/32) is due to the double counting of the exchange interaction of 4 nearest-neighbor and 4 next-nearest-neighbor atoms in the summation. Substituting ${E}_{AF{M}_{1}}-{E}_{FM}$, ${E}_{AF{M}_{{\rm{2}}}}-{E}_{FM}$ and M into the formula, we get J_1_ = 58.7 and J_2_ = 15.8 meV, respectively (See the Supplementary Information for details). During the Monte Carlo simulation, we use a 50 × 50 supercell to reduce the periodic constraints^50^. For each temperature the total number of Monte Carlo steps was 30000, allowing an initial relaxation time of 5000 steps and then sampling every 50 steps. Through Monte Carlo simulations, the variations of magnetic moment with respect to temperature are calculated. As shown in Fig. 4(d), the estimated T_C_ value is about 404 K, which is much higher than room temperature, implying that the Co_2_S_2_ monolayer has a robust ferromagnetism.

**Figure 5:**
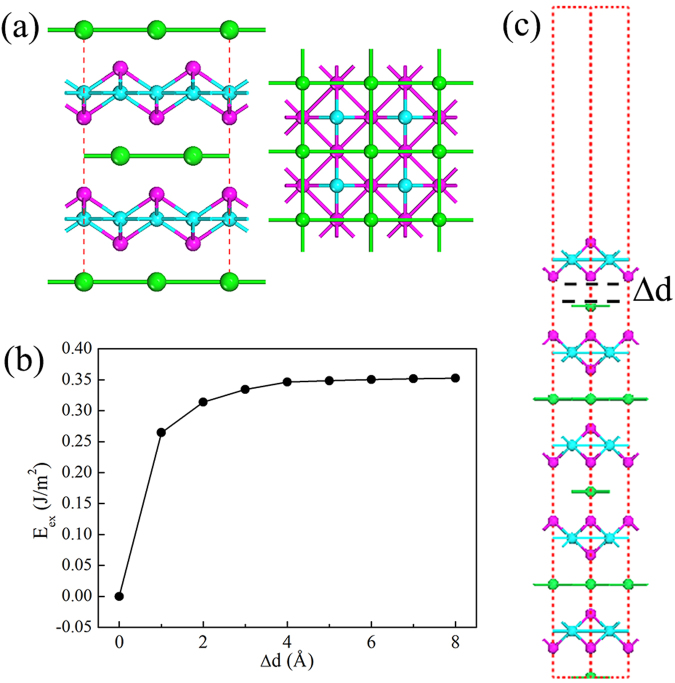
(**a**) Bulk phase of TlCo_2_S_2_, (**b**) Exfoliation energy E_ex_ as a function of the separation between the top layer and the rest of a five layered TlCo_2_S_2_ slab, (**c**) A five layered TlCo_2_S_2_ slab used to simulate the exfoliation procedure. Green, Purple and blue balls represent Tl, S and Co atoms, respectively.

Although the newly predicted 2D Co_2_S_2_ monolayer shows intriguing structural and magnetic properties for potential applications in spin-related nano-devices, how to synthesize this material is a critical issue. We note that there is a bulk material TlCo_2_S_2_^51–53^, in which the Co-S and Tl atomic layers are alternatively stacked in the z-direction, as shown in Fig. 5(a). It is interesting to note that the structure of 2D Co_2_S_2_ monolayer is exactly the same as the Co-S layer of bulk TlCo_2_S_2_. It is well-known that the monolayer MXenes can be chemically exfoliated from chemically bonded MAX phases. Hence, the Co_2_S_2_ layers could be exfoliated from the TlCo_2_S_2_ bulk by using the similar chemical exfoliation method^54^. Before measure the feasibility of exfoliating a Co_2_S_2_ monolayer from the bulk TlCo_2_S_2_, we revisit the TlCo_2_S_2_ bulk to test the quality of method that we used. The optimized lattice parameters of TlCo_2_S_2_ bulk are a = b = 3.73 Å, c = 13.00 Å, respectively. The FM configuration is found to be energetically more favorable than the AFM one by an energy difference of 155 meV, and the magnetic moment of Co atom is 0.83 μB. Our results are in good agreement with previous results^51,53^. Then we simulate the exfoliation procedure as gradually increasing the separation between the top Co_2_S_2_ layer and the rest of a five layered TlCo_2_S_2_ slab, as shown in Fig. 5(c). To estimate the exfoliation feasibility, the exfoliation energy is defined as ${E}_{ex}={E}_{C{o}_{2}{S}_{2}}+{E}_{{(TlC{o}_{2}{S}_{2})}_{4}+Tl}-{E}_{{(TlC{o}_{2}{S}_{2})}_{5}}$. During the geometry relaxation, the bottom two layers are fixed. As shown in Fig. 5(b), the total energy is seen to increase with separation Δd at first, and then slowly converges to a fixed value 0.35 J/m^2^, which is the energy (i.e. exfoliation energy E_ex_) have to be overcome under exfoliation of a Co_2_S_2_ monolayer from the bulk crystal. The calculated exfoliation energy is very close to that of the well-known graphite (0.35 J/m^2^)^55^, which directly demonstrates that a Co_2_S_2_ monolayer might be exfoliated from the bulk TlCo_2_S_2_.

## Discussion

By means of density functional theory (DFT) computations and global minimum search using particle-swarm optimization (PSO) method, we predict a new 2D cobalt sulfide monolayer, namely Co_2_S_2_, in which one Co layer is sandwiched by two S layers. Dynamical stability is predicted by the absence of any imaginary phonon modes. Molecular dynamics simulations show that this material can maintain its structural integrity at least up to 900 K. Magnetic studies and electronic structure calculations show that Co_2_S_2_ monolayer is a ferromagnetic metal with a high T_C_ up to 404 K. Finally, we propose that the Co_2_S_2_ monolayer can be synthesized be chemically exfoliated from bulk TlCo_2_S_2_ phase. Our findings greatly enrich the 2D families of transition metal sulfides.

## Methods

The first-principles calculations are performed based on the density-functional theory (DFT) implemented in Vienna ab initio simulation package (VASP)^56,57^. The exchange-correlation potential is treated in the generalized-gradient approximation (GGA) of Perdew-Burke-Eznerhof (PBE)^58^. We use the projector augmented wave (PAW) method for the description of the electron–ion interaction. The energy cutoff for the plane wave basis expansion is set to 520 eV. The k-point sampling uses the Monkhorst-Pack scheme and employs 40 × 40 × 1 and 40 × 40 × 8 mesh for Co_2_S_2_ sheet and TlCo_2_S_2_ crystal, respectively^59^. The vacuum thickness along the z axis is set 15 Å, which is enough to avoid the interaction between adjacent layers. For geometry optimization, all the internal coordinates are fully relaxed until the Hellmann-Feynman forces are less than 0.01 eV/Å. The phonon band structure of Co_2_S_2_ monolayer is calculated by using a finite displacement approach through the PHONOPY program^60^. The supercell of 4 × 4 original cell was adopted in the phonon calculation. Ab initio molecular dynamics (AIMD) simulations with canonical ensemble (NVT) at the temperature of 300, 600, 900, and 1200 K are performed with a time step of 1 fs in 5 ps, respectively. A supercell containing 4 × 4 unit cells is adopted as the model. 2D structure search is performed by using the particle-swarm optimization (PSO) method as implemented in the CALYPSO code^61,62^. During the structure search processes, the 60% structures of each generation (contains 30 structures) with lower enthalpies were selected to generate the structures for the next generation by PSO operation, and the other structures in new generation were randomly generated to increase the structural diversity. The number of generation is set to be 30. Usually, the structure searching simulation was stopped after 600~900 structures generated (20~30 generations). The ratio of cobalt and sulfur is fixed to 1:1, and the chemical formula ranges from Co_1_S_1_ to Co_4_S_4_ are considered. The local optimizations during the PSO simulation are performed using VASP. The MAE is obtained by applying the torque approach which has been proved to be an effective method for the reliable determination of MAE^63,64^. In this method, the MAE is expressed as

1 $$MAE={\sum _{i\in occ}\langle {\psi }_{i}|\frac{\partial {H}_{SO}}{\partial \theta }|{\psi }_{i}\rangle }_{\theta ={45}^{\circ }}$$

where $\theta$ is the polar angle away from the molecular axis for spin momentum, ${\psi }_{i}$ is the relativistic eigenvector, and ${H}_{SO}$ is the SOC Hamiltonian.

## Electronic supplementary material

Supplementary information
