# Supplementary material for: Two-Dimensional Co2S2 monolayer with robust ferromagnetism
Source: Sci Rep. 2017 Nov 22;7:15993. doi: 10.1038/s41598-017-16032-x (PMC5700059; doi:10.1038/s41598-017-16032-x)
Supplement: Supplementary file 1 — Supplementary information [file 41598_2017_16032_MOESM1_ESM.doc]

Supporting Information

**Two-Dimensional Co_2_S_2_ monolayer with robust ferromagnetism**

Yun Zhang^1^, Jingman Pang^2^, Meiguang Zhang^1^, Xiao Gu^3*^, Li Huang^4*^

*^1^Department of Physics and Information Technology, Baoji University of Arts and Sciences, Baoji 721016, China*

*^2^* *Department of Chemistry and Chemical Engineering, Baoji University of Arts and Science, Baoji 721016, China*

*^3^ Department of Applied Physics, Chongqing University, Chongqing 400044, P.R. China*

*^4^Department of Physics, Southern University of Science and Technology, Shenzhen, Guangdong 518055, China*

**Supplementary computational methods**

**
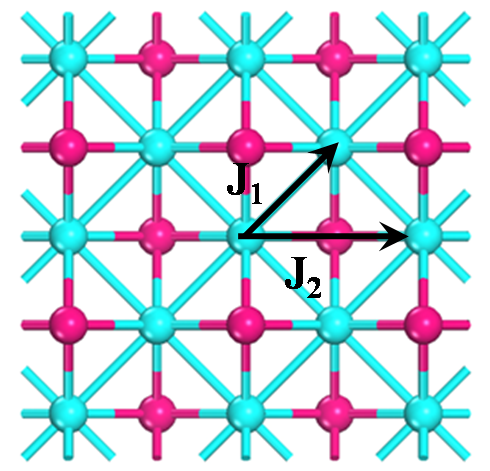
**

Figure S1 Nearest(J_1_), next-nearest(J_2_) exchange interactions of the Ising model

$$E_{FM}=E_{0}+(-16J_{1}-16J_{2})M^{2}$$

$$E_{AFM1}=E_{0}+(16J_{1}-16J_{2})M^{2}$$

$$E_{AFM2}=E_{0}+(16J_{2})M^{2}$$

So, $J_{1}=\frac{E_{AFM_{1}}-E_{FM}}{32M^{2}}$ and $J_{2}=\frac{2E_{AFM_{2}}-E_{FM}-E_{AFM_{1}}}{64M^{2}}$.
